# Neuroethics at the interface of machine learning and schizophrenia

**DOI:** 10.1038/s41537-020-0108-6

**Published:** 2020-07-20

**Authors:** Jacob McFarlane, Judy Illes

**Affiliations:** 1 Cognitive Systems, Faculty of Arts, University of British Columbia, Vancouver, BC Canada; 2 Neuroethics Canada, Division of Neurology, Department of Medicine, University of British Columbia, Vancouver, BC Canada

**Keywords:** Diseases, Psychology

## Abstract

Ethical discourse around machine learning analysis of free speech for the detection of schizophrenia has largely focused on consent and personal privacy. We focus here on additional ethics concerns and principles that must be addressed to move the pendulum of risk over to benefit and propose solutions to achieve that shift.

Recent advances in biomedicine that utilize machine learning have shown promising results in identifying psychosis through automated analysis of speech and patterns of social media use^1–4^. Indeed, in a few years, such artificial intelligence (AI) methods will lead to the possibility of predicting psychosis before a human could ever reliably do so, as well as shedding light on the underlying mechanisms of the disorder^1,5,6^. Through earlier diagnosis and treatment, these advances could be instrumental in giving lives back to individuals at risk for schizophrenia^7,8^. For all modern neurotechnological innovation, however, risks invariably parallel benefits. Do the requisite ethics exist yet to optimally support the potential benefits of AI for schizophrenia?

For many in the public, AI involvement in health care is a daunting prospect^9^. Machine learning applied in some other areas has gone awry, and biased models run the risk of perpetuating and entrenching inequities in society^10^. Machine-learning algorithms are blind to which trends arise from bias, and which reflect real differences in the world^11^. Consequently, human bias and other issues with training data can lead to biased predictions when machine-learning models are utilized^12^. Models used in Google Photos to automatically identify images, for example, labeled African Americans as gorillas^13^. A model was used by the Florida justice department for predicting the likelihood that inmates up for parole would reoffend discriminated against African-American inmates^14^. Machine-learning software was developed by Amazon for hiring new employees which systematically penalized applicants for terms related to women^15^.

Similar risks exist in using machine learning in the diagnosis of schizophrenia. Ethnic minorities and immigrants throughout the western world are diagnosed with schizophrenia more frequently than majority populations, but recent reviews suggest that these patterns do not reflect a real difference in prevalence of the disease^16^. Although the source of overdiagnosis in minorities is unclear, the consequences for machine-learning models are nonetheless troubling. Biased results from machine- learning models may further promulgate overdiagnosis and lead to misdirected treatment. Indeed, some prognostic models in development already use race as a predictor^17^.

Given past issues with machine learning and the established bias in the diagnosis of psychotic disorders, current work in pursuit of machine learning for detecting psychosis—a kind of digital phenotyping—should be focused actively on ways to reduce bias as training data sets grow and become more representative of clinical populations. The development and testing of these models have also recently raised concerns about privacy for people who live with mental illness^18,19^. While previous work has led to great strides in creating an ethical framework for these applications of such technology^10,12,20^, only limited attention has been given to how the release of models themselves or instructions on creating them might present a threat to privacy. For example, previous work has suggested that a possible future application of models that infers psychosis from prompted unstructured speech may be as part of publicly available online tools for self-assessment^21^. However, publicly accessible models will likely carry a higher risk of being misused. Unstructured speech and social media data can be easily obtained; a simple Google search grants access to twitter accounts full of possible samples. Nuanced decisions about model selection, typically thought to be technical decisions, have implications for privacy. The release of such a model or sufficiently detailed instructions for its creation threaten the privacy of not only those who choose to use it for themselves, but potentially for everyone with an online presence. These concerns must be balanced with the ethical imperatives of transparent and open research for which most frameworks for AI ethics advocate^19^. While detailed descriptions of development and testing as well as code sharing can support accountability and public trust, such practices may be harmful as these technologies begin to give fine-grained and more accurate insights about a person’s mental state than ever before, and especially if it allows third parties to develop similar models. As a recent report on AI from the EU states, “In an age of ubiquitous and massive collection of data through digital communication technologies, the right to protection of personal information and the right to respect for privacy are crucially challenged”^22^.

While general ethics frameworks may be sufficient to guide discussions about the utility and benefits of AI for psychotic disorders, academic, industry, and public cooperation will further advance discourse about its consequences, and the understanding, awareness, and solutions to the breadth of the associated technical and ethical trade-offs. Reducing the potential harms of machine learning in medicine, not the censure of innovation, should be a key part of the conversation^12,23^. Concerns for privacy—with perhaps a modernized definition of privacy for this digital age that foregrounds some form of bounded sharing over information protections and confidentiality—should inform decisions about the selection of predictor variables and the dissemination of methods. While unstructured free speech is a tool for communication that would be difficult to keep private, approaches such as federated learning may help maintain user control over their data^10^. Models need only be less biased than physicians to be an improvement over the *status quo*. Regulatory response may ultimately prove to also be a necessary element of a potential solution.

Scientists, engineers, and bioinformaticians who are pursuing machine-learning approaches for diagnosing psychosis should begin to acknowledge and engage with these ethical questions in a public setting now to preempt future issues. Ultimately, trust of patients in advances in biomedicine and the willingness to try new interventions to mitigate the burden of mental illness is key to the success of any new innovation. Dedicated conversations about the ethical issues for psychoses that so profoundly impede brain and human health will forestall harms and promote the benefits AI is intended to achieve.
